# Localized solutions of Lugiato-Lefever equations with focused pump

**DOI:** 10.1038/s41598-017-16981-3

**Published:** 2017-12-04

**Authors:** Wesley B. Cardoso, Luca Salasnich, Boris A. Malomed

**Affiliations:** 1 Instituto de Física, Universidade Federal de Goiás, 74.690-900 Goiánia, Goiás, Brazil; 2 Dipartimento di Fisica e Astronomia “Galileo Galilei” and CNISM, Universitá di Padova, Via Marzolo 8, 35131 Padova, Italy; 3 Istituto Nazionale di Ottica del Consiglio Nazionale delle Ricerche, Via Nello Carrara 1, 50019 Sesto Fiorentino, Italy; 4 Department of Physical Electronics, School of Electrical Engineering, Faculty of Engineering, Tel Aviv University, Tel Aviv, 69978 Israel; 5 ITMO University, St. Petersburg, St. Petersburg, 197101 Russia

## Abstract

Lugiato-Lefever (LL) equations in one and two dimensions (1D and 2D) accurately describe the dynamics of optical fields in pumped lossy cavities with the intrinsic Kerr nonlinearity. The external pump is usually assumed to be uniform, but it can be made tightly focused too–in particular, for building small pixels. We obtain solutions of the LL equations, with both the focusing and defocusing intrinsic nonlinearity, for 1D and 2D confined modes supported by the localized pump. In the 1D setting, we first develop a simple perturbation theory, based in the sech *ansatz*, in the case of weak pump and loss. Then, a family of exact analytical solutions for spatially confined modes is produced for the pump focused in the form of a delta-function, with a nonlinear loss (two-photon absorption) added to the LL model. Numerical findings demonstrate that these exact solutions are stable, both dynamically and structurally (the latter means that stable numerical solutions close to the exact ones are found when a specific condition, necessary for the existence of the analytical solution, does not hold). In 2D, vast families of stable confined modes are produced by means of a variational approximation and full numerical simulations.

## Introduction

It is commonly known that stable self-confined modes, such as solitons, may be produced by the balance between nonlinear and dispersive effects in the medium^1^. Solitons have been observed in diverse contexts, including water waves^2^, nonlinear fiber optics^3,4^ (as temporal solitons), Bose-Einstein condensates (BECs)^5–11^, plasmas^12,13^ and plasmonics^14,15^, proteins^16^ and DNA^17^, *etc*. Optical spatial solitons were created too in a great variety of settings, such as cells filled by vapors of alkali metals^18^, photorefractive crystals^19,20^, waveguides made of liquid dielectrics^21,22^, silica^23^ and second-harmonic-generating materials^24^, nematic liquid-crystal planar cells^25^, semiconductor waveguides^26^, arrayed waveguides^27^, and others.

While solitons have been originally introduced as exact solutions of integrable models^1,28,29^, nonintegrable systems provide for more generic and more realistic description of various physical settings. In particular, numerous dissipative systems, although lacking integrability, readily give rise to robust localized dissipative structures (LDSs), alias dissipative solitons^30,31^. In optics, an important example of a nonlinear dissipative medium which supports LDSs is provided by an optical resonator filled with a dispersive loss material featuring the Kerr nonlinearity, which is pumped by a coherent light beam (injected signal). This system is well modeled by the Lugiato-Lefever (LL) equation, originally introduced in^32^. As a mean-field equation, it applies to other settings too, such as Fabry-Perot resonators and ring cavities, fully or partially filled with nonlinear materials^32^, crystalline whispering-gallery-mode disk resonators^33^, and photonic-crystal-fiber resonators pumped by a coherent continuous-wave input beam^34,35^. In these contexts, the LL equation has been widely used to model Kerr frequency combs^36–40^, with applications to optical metrology^41^, high-precision spectroscopy^42,43^, optical atomic clocks^44,45^, phase evolution in pulse trains^46,47^, optical communications^48^, synthesis of arbitrary optical waveforms^49,50^, and radio-frequency photonics^51^. A review of the development of various applications of the LL equations has been published recently^52^. Soliton-like LDSs in the 1D LL equation are important modes too, in a broad range of values of the respective physical parameters^53^.

In many physically relevant contexts, especially as concerns realizations in optics, one- and two-dimensional (1D and 2D) LDSs supported by localized gain were studied in detail in the context of complex Ginzburg-Landau (CGL) equations^54–62^, see also reviews in^63^ and^64^. Specifically, by considering a 1D model with the tightly localized gain in the form of a delta-function, placed on top of the spatially uniform linear loss, analytical solutions for pinned LDSs pinned to the delta-function were found in^54,60^ (see also a review in^64^). Stable LDSs pinned to one or two gain-carrying “ hot spots”, shaped as narrow Gaussians, were reported too^58,62^. Further, stable 2D LDSs, including ones with an intrinsic vortex structure, supported by hot spots in the 2D geometry, were predicted in works^55–57,59,61^.

The objective of the present work is to introduce localized pump in the framework of the 1D and 2D LL equations, and find stable confined modes, which may be supported by the spatially focused pump. The difference from the previous works, which were dealing with the CGL equations^54–62,64^, is that the pump is represented by free terms in the LL equations, which do not multiply the field variable, while in the models of the CGL type the gain terms provide the parametric pump, i.e., they multiply the field variable.

We report analytical solutions of the 1D and 2D LL equation with the localized external pump, using a possibility to find exact analytical solutions for 1D modes pinned to the pump represented by the delta-function, and a variational approach, respectively. In the case of weak pump and loss, a simple perturbation theory for 1D modes is developed too. In a systematic form, the results for confined modes, including the analysis of their stability, are produced by means of numerical methods. The results demonstrate good agreement between the analytical predictions and numerical findings. In particular, while the exact analytical solutions for pinned modes in 1D are available under a special condition, we demonstrate that very similar stable numerical solutions exist when this condition does not hold. The predicted confined stable modes, pinned to the “ hot spots”, may be used, in particular, for the design of pixels placed at required positions, cf. the formation of pixels predicted by the LL equation in other contexts^65^.

## Results

### The one-dimensional Lugiato-Lefever equation. The 1D model equations

In the 1D setting, the scaled LL equation for amplitude *ϕ*(*x*, *t*) of the electromagnetic field in a nonlinear lossy cavity driven by a real localized pump *E*(*x*) is

1 $$i(\gamma +\frac{\partial }{\partial t})\varphi =[-\frac{1}{2}\frac{{\partial }^{2}}{\partial {x}^{2}}+{\rm{\Delta }}+\sigma |\varphi {|}^{2}]\varphi +E(x),$$

where *γ* > 0 is the dissipation rate, Δ is detuning of the pump with respect to the cavity, while *σ* = −1 and +1 corresponds to the self-focusing and defocusing nonlinearity, respectively. Note that dissipative solitons in the model of a fiber cavity, based on the 1D LL equation written in the temporal domain, with a pump in the form of a period train of Gaussians pulses, placed on top a nonzero background, were recently considered in work^66^. Accordingly, the LL equation (1) may also be considered in the temporal domain, with *t* and *x* replaced, respectively, by the propagation distance (*z*) and the temporal coordinate (usually denoted *τ*).

Stability of various patterns produced by Eq. (1) and its 2D counterpart considered below may be enhanced if an extra cubic lossy term, which represents the two-photon absorption, is added to the model. Then, Eq. (1) is replaced by

2 $$i(\gamma +{\rm{\Gamma }}|\varphi {|}^{2}+\frac{\partial }{\partial t})\varphi =[-\frac{1}{2}\frac{{\partial }^{2}}{\partial {x}^{2}}+{\rm{\Delta }}+\sigma |\varphi {|}^{2}]\varphi +E(x),$$

with *γ*, Γ > 0.

Before proceeding to analysis of confined modes supported by the tightly localized pump, it is relevant to mention that spatial localization may also be provided, in the presence of the usual uniform pump, by a confining (typically, harmonic-oscillator) potential^67^. On the other hand, results reported below demonstrate that the use of the narrow pump region does not imply that modes supported by it must necessarily be narrow too. Note that effects of local defects on LDSs in similar settings were previously studied in works^68,69^.

### The perturbative treatment

In the case of the self-focusing nonlinearity (*σ* = −1) and positive detuning, Δ > 0, one can develop a perturbation theory for the case of small *γ* and small *E*(*x*) in Eqs (1) and (2). In the zero-order approximation, a localized solution is given by the usual nonlinear-Schrödinger soliton^28^,

3 $$\varphi (x)={e}^{-i\zeta }\,\sqrt{2{\rm{\Delta }}}\,\text{sec}h(\sqrt{2{\rm{\Delta }}}x)$$

(as the zero-order approximation for localized patterns in the LL equation with *E* = const, the soliton waveform was used before^70^). The constant phase shift in *ansatz* (3), *ζ*, for stationary modes is then determined by the balance condition for the integral power,

4 $$P={\int }_{-\infty }^{+\infty }{|\varphi (x)|}^{2}dx\mathrm{.}$$

Indeed, it follows from condition *dP*/*dt* = 0 that

5 $$\gamma P+\Gamma {\int }_{-\infty }^{+\infty }{|\varphi (x)|}^{4}dx=-{\int }_{-\infty }^{+\infty }E(x)\text{Im}\{\varphi (x)\}dx.$$

Substituting the sech approximation (3) into Eq. (5) predicts the value of the phase shift:

6 $$\sin \,\zeta =\frac{2[\gamma +(4/3){\rm{\Gamma }}{\rm{\Delta }}]}{{\int }_{-\infty }^{+\infty }\,E(x)\,\sec \,h(\sqrt{2{\rm{\Delta }}}x)dx},$$

which is written for the generalized LL equation (2), that includes the cubic loss ∼Γ. This result makes sense if it yields |*sinζ*| ≤ 1, which implies that the LDS of the prsent type exists if the pump’s strength exceeds a threshold value, which is a combination of dissipation coefficients *γ* and Γ. In fact, a mode pinned to the localized pump exists at all values of its strength, as demonstrated by the exact solution displayed below, the threshold being an artifact following from the assumption of the rigid form of the perturbative ansatz (3).

Note that, even for $E(x)={\rm{const}}\equiv { {\mathcal E} }_{0}$, integral ${\int }_{-\infty }^{+\infty }E(x)\rm{sech} (\sqrt{2{\rm{\Delta }}}x)dx$ converges, hence the approximation based on Eqs (3)–(6) may correctly predict a state sitting on top of a small-amplitude CW background, with amplitude ${\varphi }_{0}\approx { {\mathcal E} }_{0}/(\Delta +i\gamma )$, under the condition that the LDS’s amplitude, (2Δ)^1/2^, is much larger than *ϕ*_0_, i.e., ${ {\mathcal E} }_{0}^{2}\ll {{\rm{\Delta }}}^{3}$. Detailed comparison of predictions of the perturbation theory with numerical results will be presented elsewhere.

### A particular exact solution and states close to it

In the case when the gain is localized in a very narrow region, it may be approximated by the Dirac’s delta-function, cf. a similar approximation adopted for a strongly localized gain in the CGL model^54^:

7 $$E(x)={E}_{0}\,\delta (x)$$

(a similar model including a localized gain, with an LDS pinned to it, was also formulated in terms of the Swift-Hohenberg equation^71^). This means that the homogeneous version of Eq. (2),

8 $$i(\gamma +{\rm{\Gamma }}|\varphi {|}^{2}+\frac{\partial }{\partial t})\varphi =[-\frac{1}{2}\frac{{\partial }^{2}}{\partial {x}^{2}}+{\rm{\Delta }}+\sigma |\varphi {|}^{2}]\varphi ,$$

must be solved with the boundary condition at *x* = 0 which determines the jump of the first derivative induced by *δ*(*x*) in Eq. (7):

9 $$\frac{d\varphi }{dx}{|}_{x=+0}-\frac{d\varphi }{dx}{|}_{x=-0}=2{E}_{0}.$$

In this case, one can find a particular exact solution to the generalized LL equation (8) in the form of

10 $$\varphi (x)=\frac{A{e}^{i\zeta }}{{[\sinh (\lambda (|x|+\xi ))]}^{1+i\mu }},$$

with parameters (*μ* is called the *chirp*)

11 $$\mu =-\gamma /{\lambda }^{2},$$

12 $${A}^{2}=3\gamma /(2{\rm{\Gamma }}),$$

13 $${\lambda }^{2}=\frac{\gamma }{4\Gamma }(\sqrt{9{\sigma }^{2}+8{{\rm{\Gamma }}}^{2}}+3\sigma ).$$

This particular solution is a non-generic one, as it exists at the *single value* of the mismatch parameter,

14 $${\rm{\Delta }}=\frac{{\lambda }^{2}}{2}(1-{\mu }^{2})\equiv \frac{1}{2}(\frac{3\sigma \gamma }{{\rm{\Gamma }}}-{\lambda }^{2})$$

(in other words, it is a *codimension-one* type of the exact solution, with “ one” referring to constraint (14), which must be adopted to produce the analytical expression). Note that the solution given by Eq. (13) exists (i.e., it gives *λ* ^2^ > 0) for both *σ* = −1 and +1. The presence of the cubic-loss coefficient, Γ > 0, is necessary for the existence of the solution. Indeed, in the limit of Γ → 0 Eq. (13) leads to divergence:

15 $${\lambda }^{2}\approx \{\begin{array}{c}3\gamma /(2{\rm{\Gamma }})\,{\rm{at}}\,\sigma =+1,\\ (1/3){\rm{\Gamma }}\gamma \,{\rm{at}}\,\sigma =-1.\end{array}$$

Finally, parameters *ξ* and *ζ* in expression (10) are obtained by its substitution in jump condition (9):

16 $$A\lambda (1+i\mu ){e}^{i\zeta \lambda }\frac{\cosh (\lambda \xi )}{{[\sinh (\lambda \xi )]}^{2+i\mu }}=-{E}_{0}.$$

An explicit result, following from Eq. (16), is

17 $$\xi =\frac{1}{2\lambda }{\rm{arcosh}}(1+\frac{\chi }{{E}_{0}^{2}}+\sqrt{4+\frac{\chi }{{\lambda }^{2}{E}_{0}^{4}}}),$$

$$\chi \equiv {A}^{2}{\lambda }^{2}(1+{\mu }^{2}),$$

18 $$\zeta =\pi -\arctan \,\mu +\mu \,\mathrm{ln}(\sinh (\lambda \xi )),$$

where arcosh(*Z*) ≡ *ln*(*Z* + (*Z* ^2^ −1)^1/2^).

In the CGL model with localized gain (rather than pump), exact pinned states are also codimension-one solutions, the difference being that, in the latter case they coexist with the zero state, which may or may not be stable solutions^54^.

**Figure 1:**
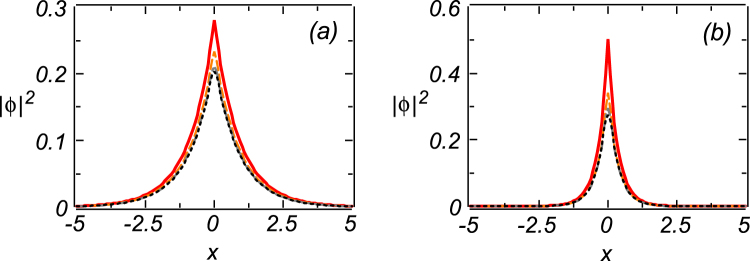
Solid red lines display the exact solution (10) for the mode pinned to the delta-functional pump, and a set of numerical solutions based on the use of the regularized delta-function defined as per Eq. (31) (see Methods), with *w* = 0.05 (dashed orange lines), *w* = 0.1 (dashed-dotted gray lines), and *w* = 0.15 (dotted black lines). The results presented in (**a**) and (**b**) pertain to self-focusing (*σ* = −1) and self-defocusing (*σ* = 1) signs of the nonlinearity, respectively. All these solutions are stable. Other parameters are *E*_0_ = *γ* = Γ = 1, while Δ is given by Eq. (14).

In Fig. 1(a,b) we display typical examples of the analytically found modes pinned to the delta-function for focusing and defocusing nonlinearities, by choosing *σ* = −1 and *σ* = 1, respectively, along with their numerically found counterparts. In this case, we set parameters as *γ* = Γ = *E*_0_ = 1, and took Δ as per Eq. (14). The numerical counterparts were produced by using the naturally regularized delta function in the form given by Eq. (31) (see Methods), for three different values of width *w*. It is relevant to mention that, while the regularized delta-function approaches the standard delta-function in the limit of *w* → 0, the use of a finite stepsize Δ*x* in the numerical procedure gives rise to a critical value ${w}_{cr}\simeq {\rm{\Delta }}x/2$ of *w*, the numerical solution getting drastically different from the analytical one at *w *< *w*_cr_. With the increase of the cubic-loss strength, Γ, *w*_cr_ blows up (increases very fast) at $\Gamma \gtrsim 3$.

**Figure 2:**
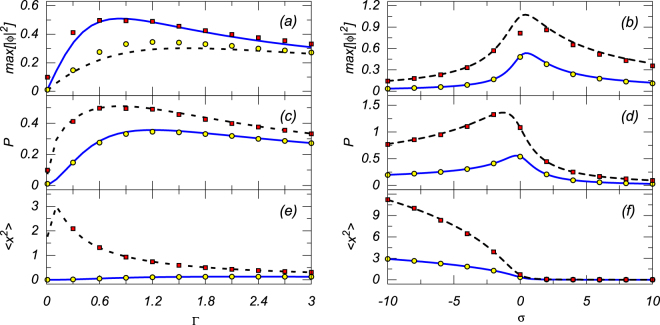
Panels (a,b), (c,d), and (e,f) show, severally, the peak local power, max[|*ϕ*|^2^], integral norm *P* (see Eq. (4)), and the mean squared width 〈*x* ^2^〉 (see Eq. (19)) of the analytical mode (10) versus the cubic-loss and self-interaction strengths, Γ and *σ* (left and right columns). The left columns correspond to *γ* = *E*_0_ = 1 and *σ* = 1 (solid blue lines) or *σ* = −1 (dashed black lines), i.e., the self-defocusing and focusing, respectively. In the right columns we set *γ* = Γ = 1 and *E*_0_ = 1 (solid blue lines) or *E*_0_ = 2 (dashed black lines). The corresponding numerical results are shown by chains of yellow circles and red boxes, respectively. The numerical data displayed here and other figures have been produced using the regularized delta-function (31), with *w* close to its above-mentioned critical value (see Methods).

Further, in Fig. 2 we present systematic results for the 1D modes produced by analytical solution (10) and its numerical counterparts. These are the peak local power, max[|*ϕ*|^2^], the integral power, *P* (see Eq. (4)), and the mean squared width,

19 $$\langle {x}^{2}\rangle ={P}^{-1}{\int }_{-\infty }^{+\infty }{x}^{2}{|\varphi (x)|}^{2}dx,$$

shown in the left and right panels of the figure, as functions of the two nonlinearity coefficients, *viz*., the cubic-loss strength Γ and self-interaction strength *σ* (in the right panel, *σ* is considered as a continuously varying parameter, while in the left panel it is fixed to be *σ* = ±1 for the self-defocusing and focusing cases). Note that, as predicted by the analytical solutions (see Eqs (10) and (15)), the integral power *P* vanishes at Γ → 0. On the other hand, the results pertaining to *σ* = +1 and −1 tend to converge at large values of Γ, as the dissipative nonlinearity is dominant in this limit. The numerically generated findings are very close to the analytical predictions.

It is worthy to note conspicuous maxima of the peak local power and integral power, observed in Fig. 2(b) at *σ* = 0 and *σ* ≈ −1, respectively. Further, the bottom panel in Fig. 2(b) reveals a *counter-intuitive* feature of the pinned states: they shrink (〈*x* ^2^〉 → 0) in the limit of large *σ* > 0, i.e., *strong self-defocusing* (the same is also demonstrated by Eq. (13), which predicts ${\lambda }^{2}\sim \mathrm{1/}\langle {x}^{2}\rangle \to \infty$ at *σ* → +∞). Usually, self-confined modes shrink in the opposite limit, of strong self-focusing. This surprising finding may be explained by the effect introduced by the cubic loss term ~Γ. Indeed, as mentioned above, the exact solution for the pinned state does not exist at Γ = 0, and, in the presence of Γ > 0, the shape of the mode is essentially affected by its chirp, which is produced by Eq. (11).

**Figure 3:**
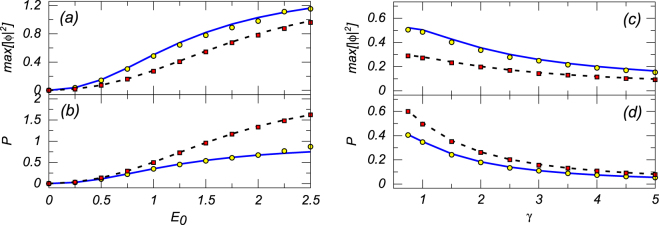
Panels (a,c) and (b,d) show the peak local power (max[|*ϕ*|^2^]) and integral norm (*P*, see Eq. (4)), respectively, versus the pumping amplitude *E*_0_ and the dissipation rate *γ* (left and right columns). The left columns correspond to *γ* = Γ = 1 while in the right columns we set *E*_0_ = Γ = 1, both with *σ* = +1 and *σ* = −1, i.e., the self-defocusing and focusing (the corresponding analytical results are displayed by solid blue lines and dashed black lines, respectively), while the corresponding numerical results are shown by chains of yellow circles and red boxes, respectively.

Further, Fig. 3 displays the effect of the variation of the pump’s amplitude *E*_0_ and dissipation coefficient *γ* on the peak local power (max[|*ϕ*|^2^]) and integral power *P* (see Eq. (4)) of numerical solutions obtained from Eq. (2), for both the self-defocusing and focusing signs of the nonlinearity, i.e., *σ* = +1 and *σ* = −1, respectively, along with the counterparts predicted by the above analytical solutions. Naturally, the peak local and integral powers increase with the growth of *E*_0_, and decrease with the growth of *γ*. These properties can be used for an effective control of the localized modes by means of parameters *E*_0_ and *γ*.

Comparing the results obtained for the self-defocusing (*σ* = +1) and self-focusing (*σ* = −1) signs of the nonlinearity, we again observe a “ counter-intuitive” phenomenon, similar to that mentioned above, i.e., the solution is more localized in the case of the self-defocusing case than in the self-focusing case. Note that numerical results closely follow their analytical counterparts in Fig. 3 too.

**Figure 4:**
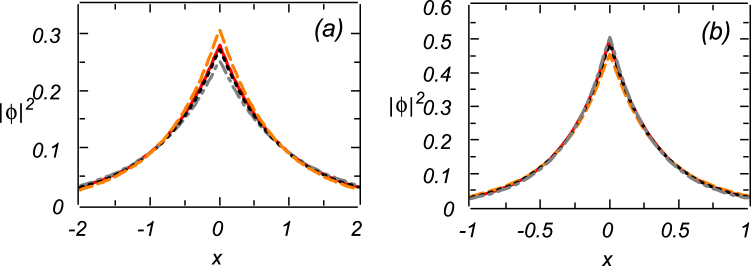
Solid red lines display the exact solution (10) for the mode pinned to the delta-functional pump. They are compared to a set of numerically generated solutions produced with the help of the regularized delta-function: dotted black lines pertain to mismatch parameter Δ taken exactly as per Eq. (14); dashed orange lines pertain to Δ → 0.75Δ, and dashed-dotted gray lines pertain to Δ → 1.25Δ. The results displayed in panels (a) and (b) are obtained for the self-focusing (*σ* = −1) and self-defocusing (*σ* = +1) signs of the nonlinearity, respectively. All these solutions are stable. Other parameters are *E*_0_ = *γ* = Γ = 1.

Because the above codimension-one analytical solution is valid only under condition (14) imposed on the parameters, it is necessary to investigate the *structural stability* of the pinned modes against departure from this condition. To this end, in Fig. 4 we compare the solutions (both analytical and numerically found ones) obtained with the value of Δ selected as per Eq. (14), and their numerical counterparts obtained with this Δ replaced by 0.75Δ and 1.25Δ. We conclude that these considerable variations of Δ produce a weak effect on the solutions, i.e., they are structurally stable, effectively representing generic pinned modes, rather than specially selected ones.

While changes in the profiles of the solutions produced by the variation of Δ are relatively small, it is relevant to mention that the solutions are more sensitive to the variation in the case of the self-focusing than in the defocusing cas**e**.

Finally, systematic simulations of the perturbed solutions corroborate the stability of all the numerical solutions emulating the analytically predicted modes pinned to the delta-function. In fact, all the solutions are strong *attractors*, as direct simulations demonstrate that Eq. (2) readily generates precisely these states, starting from the zero input, *ϕ*(*x*, 0) = 0. This numerical result is important, because the stability of the analytically found solutions cannot be explored in an analytical form.

### The two-dimensional Lugiato-Lefever equation

The 2D version of the 1D LL equation (1) is

20 $$i(\gamma +\frac{\partial }{\partial t})\varphi =[-\frac{1}{2}{\nabla }_{\perp }^{2}+{\rm{\Delta }}+\sigma |\varphi {|}^{2}]\varphi +E(x,y),$$

where ${\nabla }_{\perp }^{2}=\frac{{\partial }^{2}}{\partial {x}^{2}}+\frac{{\partial }^{2}}{\partial {y}^{2}}$, and the cubic loss is not included here (Γ = 0), as, unlike the exact 1D solutions, this term is not necessary for finding 2D solutions reported here. Further, one may fix here *γ* = 1 by means of rescaling. Below, we consider the Gaussian 2D shape of the pump, given by

21 $$E(x,y)=\frac{{P}_{0}}{\sqrt{\pi }\eta }{\rm{e}}{\rm{x}}{\rm{p}}(-\frac{{x}^{2}+{y}^{2}}{2{\eta }^{2}}),$$

where *P*_0_ is the pump’s integral intensity, and parameter *η* controls its width.

**Figure 5:**
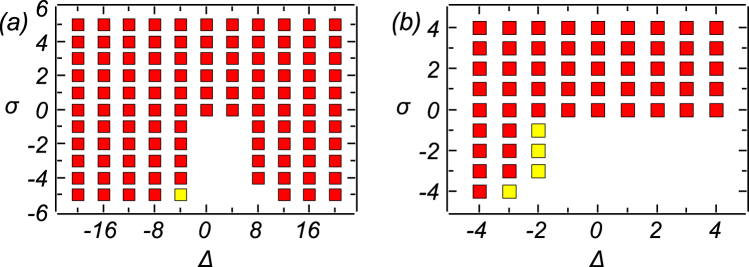
Existence diagrams for stable 2D modes in the plane of parameters (Δ, *σ*), as produced by direct simulations of Eq. (20), with pump’s parameters *P*_0_ = 10 and *η* = 1. Panel (a) covers the range of *σ* ∈ [−5, 5] and Δ ∈ [−20, 20], while (**b**) is a zoom of (**a**) for *σ* ∈ [−4, 4] and Δ ∈ [−4, 4]. The region covered by red boxes is populated by single-peak (bell-shaped) modes (see Fig. 7), while yellow boxes designate parameters at which the shape of the modes is crater-shaped, featuring the maximum local power at a finite difference from the center, see an example in Fig. 9 below.

In Fig. 5 we display the existence diagram of stable solutions produced by direct simulations of Eq. (20) in the plane of the mismatch and nonlinearity coefficients, (Δ, *σ*), for fixed pump’s parameters, *P*_0_ = 10 and *η* = 1. Light yellow boxes denote values of parameters at which stable 2D solutions are crater-shaped (see Fig. 9 below), while red boxes correspond to the single-peak (bell-shaped) solutions, as shown below in Fig. 7. As concerns variational equations (28)–(30) (see Methods), their physically relevant solutions, corresponding to *B* > 0, have been found, by means of the relaxation method, for all values of parameters covering the range of *σ* ∈ [−5, 5] and Δ ∈ [−20, 20], the respective picture essentially coinciding with one displayed on the basis of the full numerical solution in Fig. 5(a).

**Figure 6:**
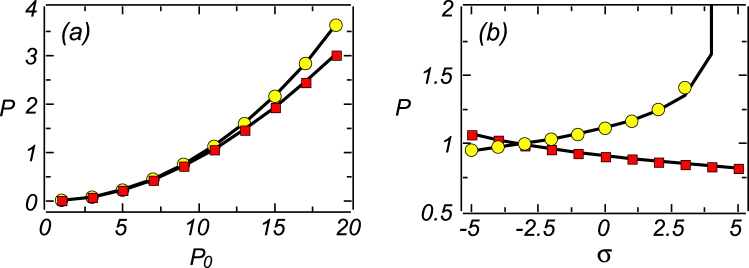
(**a**) The integral power of the 2D modes, *P*, *vs* the pump’s amplitude, *P*_0_, for Δ = 10 and *σ* = −1, shown by the line with yellow circles, and *σ* = +1, the line with red boxes. (**b**) The integral power *vs σ*, pertaining to *P*_0_ = 10 and Δ = −10 or Δ = +10, shown by lines with yellow circles or red boxes, respectively. Numerical and variational solutions are indistinguishable in the range shown in the plots. Other parameters are *γ* = *η* = 1.

In Fig. 6(a) and (b) we display the integral power of the confined 2D modes, *P*, defined as per Eq. (25), as a function of the pump’s amplitude *P*_0_ and nonlinearity strength *σ*, respectively (see Eq. (21)). Note that in Fig. 6(a) the analytical results, produced by the variational *ansatz* (26), and their numerical counterpart, obtained from direct simulations of Eq. (20), are very close to each other. We observe that, in the self-focusing case (*σ* = −1, shown by the line with circles), the integral power is slightly larger than in the self-defocusing case (*σ* = 1, shown by the line with boxes). In Fig. 6(b), the abrupt growth of the power for Δ = −10 at *σ* > 4 make the numerical solutions unstable.

**Figure 7:**
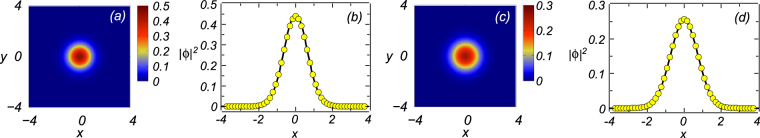
Profiles of stable 2D modes, |*ϕ*(*x*, *y*)|^2^, as produced by direct simulations of Eq. (20) (at *t* = 100), for (**a**) Δ = −10 and (**c**) Δ = 10. Displayed in panels (b) and (d) are transverse profiles, |*ϕ*(*x*, 0)|^2^, corresponding to the 2D shapes shown in (**a**) and (**c**), respectively. Lines in (**b**) and (**d**) depict the approximate analytical solution based on *ansatz* (26), while chains of yellow circles represent the numerical solution. Other parameters are *P*_0_ = 10 and *σ* = *γ* = *η* = 1.

Generic examples of the local-power profiles, |*ϕ*|^2^, for the 2D modes, obtained from direct simulations (at *t* = 100) for two different values of Δ, and the comparisons with the corresponding approximate analytical solutions, based on *ansatz* (26), are displayed in Fig. 7. Actually, the numerical and analytical profiles are indistinguishable at these values of Δ, in accordance with the above results which also demonstrated very good agreement of the analytical predictions with the numerical counterparts at large values of Δ. However, at small values of Δ, the numerical solutions feature a strong increase in the norm and may become unstable. In this case, the analytical approximation is not relevant.

**Figure 8:**
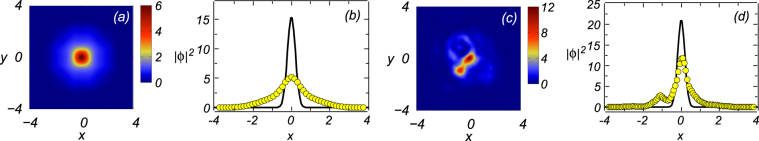
Profiles of 2D solutions, |*ϕ*(*x*, *y*)|^2^, and the corresponding transverse profile, |*ϕ*(*x*, 0)|^2^, for Δ = −1 in (**a**), (**b**), and Δ = +1 in (**c**), (**d**). In (**b**) and (**d**), solid black lines represents the analytical results, that were used as inputs for the direct simulations. Results of the simulations (at *t* = 10) are shown by yellow circles. Other parameters are *σ* = −1, *P*_0_ = 10, and *γ* = *η* = 1.

The situation in a parameter region where stable stationary modes are absent (see Fig. 5) is illustrated by numerically generated solutions (at *t* = 10) displayed in Fig. 8(a,b) for *σ* = −1 and Δ = −1, and in Fig. 8(c)–(d) for *σ* = −1 and Δ = +1. Due to the instability of the numerical solutions, the analytical predictions are not relevant in this case.

**Figure 9:**
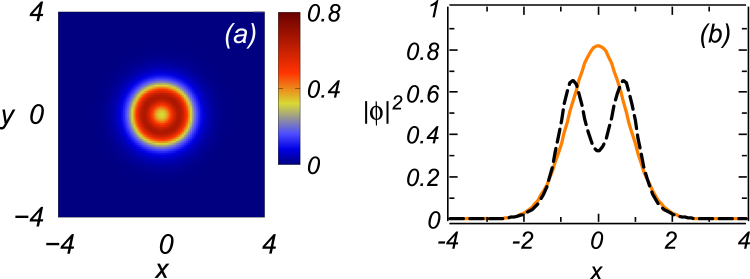
(**a**) The same as in Fig. 7(a), but with *σ* = −5 and Δ = −4. (**b**) The black dashed line is the static profile, |*ϕ*(*x*, 0)|^2^, of the numerically generated crater-shaped mode (at *t* = 100). The orange solid line shows a formal prediction of the variational approximation for these values of the parameters.

As mentioned above, in a small part of their existence region (covered by yellow squared in Fig. 5), numerically found stable 2D modes feature a crater-like shape, with the maximum of the local power attained at a finite difference from the center, see an example in Fig. 9. Obviously, the analytical approximation based on *ansatz* (26) cannot reproduce this shape.

## Discussion

The modifications of the well-known 1D and 2D LL (Lugiato-Lefever) equation introduced in this work, with tightly localized pump, make it possible to create new stable confined modes, which are of interest in terms of the use of the LL equations as models of the pattern formation in nonlinear dissipative media. They may also be used to design compact pixels that can be created in cavities modeled by the LL equations. The present work is based on the combination of analytical and numerical methods, in the 1D and 2D geometries alike, the analytical parts helping to achieve a deeper insight into the variety of steady-state confined modes produced by the LL equations.

In the 1D geometry, we have first developed a simple perturbation theory, based on the usual sech *ansatz* (3), in the case of weak pump and loss. Other results have produced a family of exact analytical solutions, assuming that the tightly focused gain is represented by the delta-function, while the self-interaction may have both focusing and defocusing signs (*σ* < 0 and *σ* > 0, respectively). The analytical form of the solution is given by Eqs (10)–(13), under the condition that the mismatch, Δ, takes the specially selected value (14), and the cubic nonlinear term, which represents two-photon losses in the optical medium (with rate Γ), is present. Furthermore, numerical results, displayed in Fig. 4, corroborate the structural stability of the codimension-one analytical solutions, because the deviation of Δ from the spacial value (14) leads to weak variation of the stable pinned solutions. Most essential parameters which control the shape of the 1D pinned modes are two nonlinearity coefficient, *σ* and Γ. Characteristic features of the solution is the cusp at the center, and the phase structure (chirp). A remarkable fact is that the exact solutions are very close to their numerical counterparts, produced by the localized pump shaped as a regularized delta-function, and the family of the so generated 1D modes is entirely stable. In fact, the proximity of the numerical and analytical solutions additionally confirms the structural stability of the latter. A noteworthy (and counter-intuitive) feature of the 1D modes is that they *shrink* with the increase of the strength of the self-defocusing nonlinearity. The 1D solution produced by the analysis may help to find similar states in more general pattern-formation models.

In the 2D geometry with the pump applied at a small Gaussian-shape “ hot spot”, systematic numerical results are reported in the combination with approximate analytical findings produced by the variational approximation. A vast stability area in the system’s parameter space has been found, the most essential parameters being the above-mentioned mismatch and nonlinearity coefficients, Δ and *σ* (the 2D system is considered without the two-photon loss, Γ = 0, as its presence is not a necessary condition for finding the relevant solutions). In most cases, the 2D modes pinned to the “ hot spot” feature a single-peak (bell-shaped) structure, which is stable, and is well approximated by the variational *ansatz*. In a small part of the parameter space, 2D stable modes feature a crater-like shape, with the maximum local power found at a finite distance from the center. In another small part of the parameter space, 2D modes are unstable.

As an extension of the analysis, it may be interesting to use numerical methods to construct modes pinned to a set of two mutually symmetric 1D or 2D hot spots, cf. a similar configuration elaborated for the 1D CGL equation in ref.^72^. In particular, in the case of the self-focusing nonlinearity, *σ* < 0, one may expect spontaneous symmetry breaking between peaks attached to the two pump maxima. On the other hand, in the 2D geometry it may also be interesting to introduce ring-shaped pump, which may give rise to confined modes with a vortex structure, cf. a similar consideration for the 2D CGL equation in ref.^61^. A possibility of spontaneous breaking of the axial symmetry in vortex modes may be addressed too, following the pattern of the analysis performed in the framework of the CGL equation^74^.

## Methods

### The variational approach

Firstly, we define *ϕ*(*x*, *y*, *t*) ≡ Φ(*x*, *y*, *t*) exp (−*γt*), casting Eq. (20) in the form of

22 $$i\frac{\partial }{\partial t}{\rm{\Phi }}=[-\frac{1}{2}{\nabla }_{\perp }^{2}+{\rm{\Delta }}+\sigma {e}^{-2\gamma t}|{\rm{\Phi }}{|}^{2}]{\rm{\Phi }}+E{e}^{\gamma t},$$

which can be derived from a real time-dependent Lagrangian,

23 $$\begin{array}{rcl}L & = & \int \int dxdy\{\frac{i}{2}({{\rm{\Phi }}}_{t}^{\ast }{\rm{\Phi }}-{{\rm{\Phi }}}^{\ast }{{\rm{\Phi }}}_{t})+\frac{1}{2}(|{{\rm{\Phi }}}_{x}{|}^{2}+|{{\rm{\Phi }}}_{y}{|}^{2})\\  &  & +\Delta |{\rm{\Phi }}{|}^{2}+\frac{\sigma }{2}{e}^{-2\gamma t}|{\rm{\Phi }}{|}^{4}+E{e}^{\gamma t}({{\rm{\Phi }}}^{\ast }+{\rm{\Phi }})\}\,\mathrm{.}\end{array}$$

Note that the following exact power-balance equation is produced by Eq. (20):

24 $$\frac{dP}{dt}=-2\gamma P-2\iint \text{Im}\{\varphi (x,y,t)\}E(x,y)dxdy,$$

for the integral power defined as

25 $$P=\int \int |\varphi (x,y,t){|}^{2}dxdy,$$

cf. the 1D counterpart given by Eq. (5).

For the variational approximation, we use the 2D isotropic Gaussian *ansatz* ^73^,

26 $${\rm{\Phi }}={e}^{\gamma t}A(t)\exp [-(B(t)-iC(t))({x}^{2}+{y}^{2})],$$

where *A*, *B* and *C* are real variational parameters, subject to obvious constraint *B* > 0. Next, substituting the *ansatz* in Eq. (23) and performing the integration, we arrive at the following effective Lagrangian:

27 $$\begin{array}{rcl}{L}_{{\rm{eff}}} & = & \frac{\pi }{2}{e}^{2\gamma t}\{\frac{{A}^{2}}{2{B}^{2}}\frac{dC}{dt}+[1+\frac{{\rm{\Delta }}}{B}+\frac{{C}^{2}}{{B}^{2}}]{A}^{2}\\  &  & +\frac{\sigma {A}^{4}}{4B}+\frac{8\eta {P}_{0}(1+2B{\eta }^{2})A}{1+(4{B}^{2}+4{C}^{2}){\eta }^{4}+4B{\eta }^{2}}\}.\end{array}$$

The variational (Euler-Lagrange) equations following from Lagrangian (27), ∂*L*_eff_/∂(*A*, *B*, *C*) = 0, are

28 $$\sigma {A}^{3}+\frac{[4B(B+{\rm{\Delta }})+4{C}^{2}]}{2B}A+\frac{8\eta {P}_{0}{B}^{2}(1+2B{\eta }^{2})}{\sqrt{\pi }[4{B}^{3}{\eta }^{4}+4{B}^{2}{\eta }^{2}+(4{\eta }^{4}{C}^{2}+1)B]}=0,$$

29 $$\sigma B{A}^{3}+4({\rm{\Delta }}B+2{C}^{2})A+\frac{8{\eta }^{3}{P}_{0}{B}^{3}[1+4({B}^{2}-{C}^{2}){\eta }^{4}+4B{\eta }^{2}]}{\sqrt{\pi }{(1+4({B}^{2}+{C}^{2}){\eta }^{4}+4B{\eta }^{2})}^{2}}=0,$$

30 $$\frac{\pi (2C-\gamma )A}{2{B}^{2}}-\frac{32{\eta }^{5}{P}_{0}(1+2B{\eta }^{2})C}{\sqrt{\pi }{[1+4({B}^{2}+{C}^{2}){\eta }^{4}+4B{\eta }^{2}]}^{2}}=0.$$

### Numerical simulations

To solve the one-dimensional equation (2) numerically, we made use of the regularized delta-function based on the usual Gaussian expression (see ref^64^. and references therein):

31 $$\tilde{\delta }(x)={(\sqrt{\pi }w)}^{-1}\exp (-{x}^{2}/{w}^{2}),$$

with finite width *w*.

We employed a fourth-order split-step method to solve Eqs (2) and (20), starting from the zero input, *ϕ*(*x*, 0) = 0. An output was categorized as a stable mode if it maintained a static profile for a long time ($ts\sim 1000$, which corresponds $\gtrsim 100$ characteristic diffraction times). In most simulations, the spatial and temporal steps were fixed as Δ*x* = 0.04 and Δ*t* = 0.001. To produce the numerical results for the 1D LL equation, shown in Fig. 2, we chose values of *w* such that the resultant integral power was different from the analytical counterpart, corresponding to exact solution (10), by no more than 3%.

To solve Eqs (28)–(30) for *A*, *B*, and *C*, produced by the variational approximation, we used a relaxation method with a fixed error constraint of 10^−6^. Then, the so found values were inserted in *ansatz* (26) to produce the full variational approximation for the 2D modes. Lastly, the above-mentioned scaled value of the dissipation parameter, *γ* = 1, was set in all the simulations.
